# Anaesthesia Management in a Case of Ludwig’s Angina With Difficult Airway Managed by Emergency Tracheostomy

**DOI:** 10.7759/cureus.66597

**Published:** 2024-08-10

**Authors:** Aparna Bagle, Sanya Varma, Subha Teresa Jose Vazhakalayil

**Affiliations:** 1 Anaesthesiology, Dr. D. Y. Patil Medical College, Hospital and Research Centre, Dr. D. Y. Patil Vidyapeeth (Deemed to be University), Pune, IND

**Keywords:** airway obstruction, ludwig's angina, difficult airways, anesthesiologists, neck infections

## Abstract

Severe neck infections present significant challenges for anesthesiologists due to the complexities associated with managing difficult airways. Ludwig's angina, a rapidly progressing infection of the submandibular space, exemplifies these challenges due to the high risk of airway obstruction. This case report details an emergency procedure performed to drain Ludwig's angina, highlighting the difficulties encountered and the strategies employed. Awake fiberoptic intubation is demonstrated as an effective approach for maintaining the airway during such operations. The report underscores the critical nature of quick and effective management, emphasizing the importance of readiness for interventions such as tracheostomy in cases where oxygen saturation drops, thereby ensuring patient safety in precarious situations.

## Introduction

Ludwig's angina is a high-risk condition that can be potentially fatal. It is characterized by gangrenous cellulitis of the neck, which spreads through the continuity of the fascial planes. This can lead to edema, obstruction, or distortion of the airway, necessitating prompt treatment. The usual approach involves aggressive antibiotic therapy and, in many cases, surgical drainage, with a critical emphasis on protecting the airway at all costs [1].

Ludwig's angina may present with bilateral cervical swelling, dysphagia, drooling, neck tenderness, elevation and posterior displacement and swelling of the tongue, restricted neck movements, trismus, dyspnea, and stridor, all of which can complicate airway management [2]. Therefore, it is essential to thoroughly assess the patient preoperatively to identify features that may cause difficulties with mask ventilation, direct laryngoscopy, and intubation. Alternative methods of ventilation should be considered and readily accessible, along with a clear plan for their use if mask ventilation or intubation proves impossible [3].

## Case presentation

A 64-year-old male patient, previously in good health, presented with progressive pain and swelling in the submandibular region over the past two weeks. The patient had a molar tooth extraction two weeks prior and had a history of ischemic heart disease, having undergone percutaneous transluminal coronary angioplasty with a stent placed in the right coronary artery three years ago. He had been on treatment for hypertension for two years, taking Tab. Amlodipine 5 mg, Tab. Ecosprin 150 mg, Tab. Metoprolol 25 mg, and Tab. Clopidogrel 75 mg. He had stopped taking Clopidogrel five days before the scheduled surgery.

**Figure 1:**
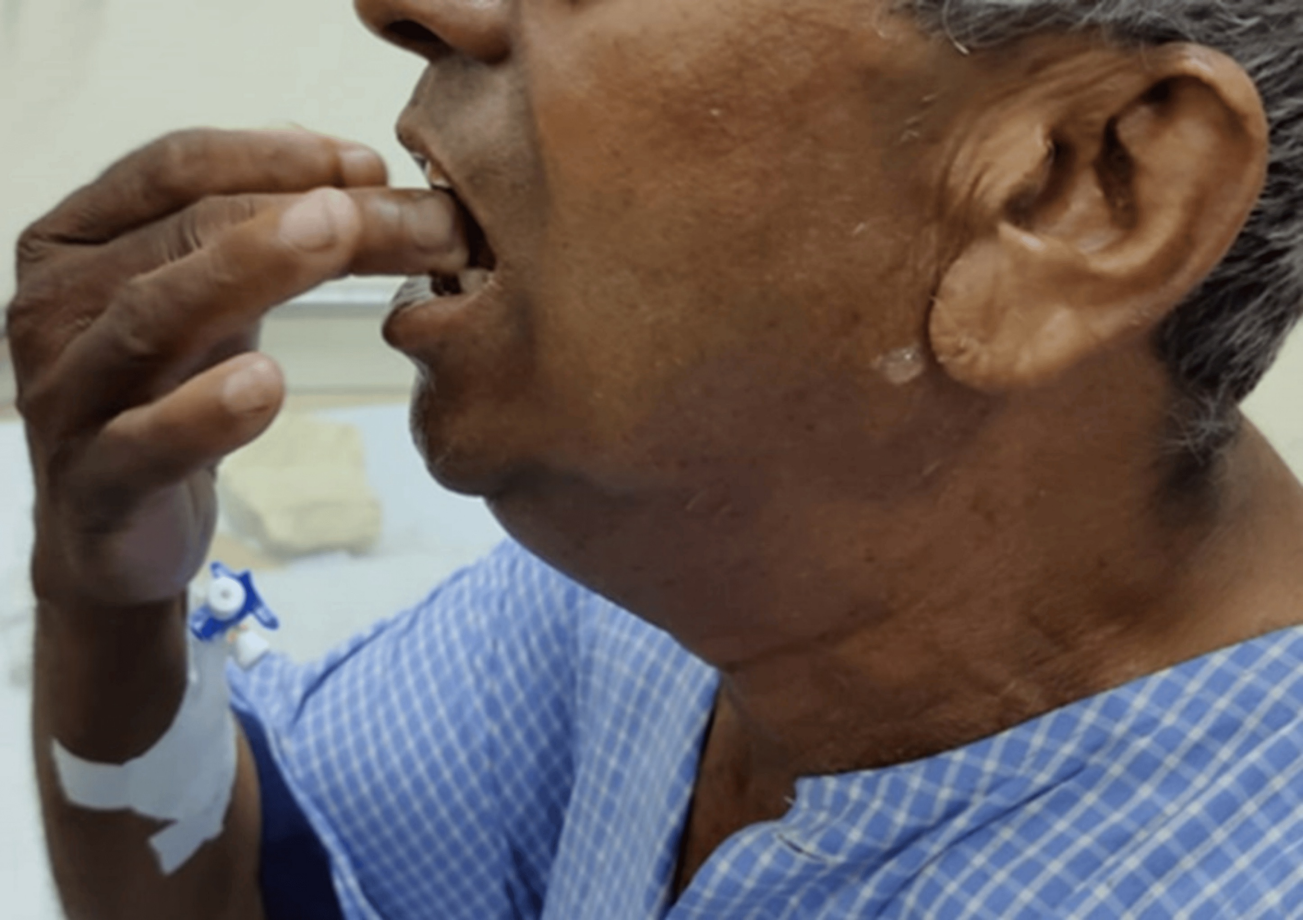
Pre-operative assessment of airway with mouth opening 1 finger width

**Figure 2:**
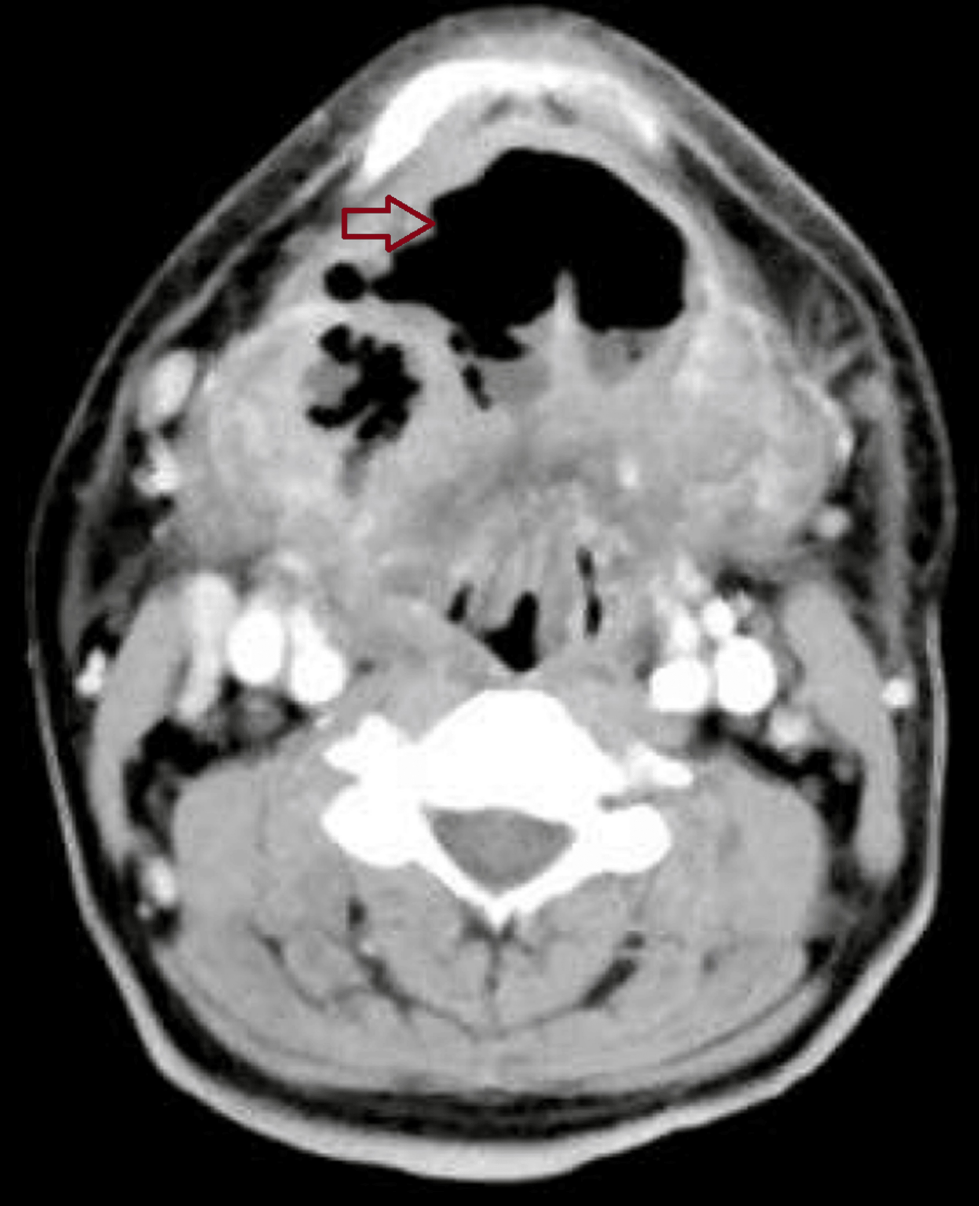
Contrast CT Neck Thickening and increased attenuation diffusely on the floor of the mouth, submandibular space. Associated gas collection on the floor of the mouth and bilateral cervical lymphadenopathy. CT=computed tomography

A preoperative assessment revealed a difficult airway: the patient’s mouth opening was limited to one finger width (Figure 1), with a Mallampati classification (MPC) score of 4, restricted temporomandibular joint movement, a short neck, heavy jaw, double chin, and a thyromental distance of less than 6 cm (less than three finger breadths), with multiple missing teeth. A two-dimensional echocardiography (2D-Echo) showed normal results. Contrast CT neck revealed an ill-defined hypodense 32×24×16mm lesion in the submandibular region, suggestive of submandibular abscess with gas formation (Figure 2).

The patient was preoperatively informed about the need for awake intubation and provided high-risk, intensive care unit (ICU), and ventilator consent. Bilateral nostril patency was confirmed using a spatula with observed fogging. After obtaining a difficult airway consent, the patient received an injection (inj) of Glycopyrrolate 0.2 mg intramuscularly (IM), an 18G cannula was secured, and nasal packing with 2% lignocaine and the adrenaline was performed 20 minutes before surgery. Oxymetazoline hydrochloride nasal drops were applied, and nebulization with 4% lignocaine 2 ml, 20 mcg inj. of Dexmedetomidine, and 3 ml normal saline was administered.

In the operating room, all monitors were attached, and consents were rechecked. A difficult airway cart was prepared, including a fibreoptic bronchoscope, Cardiac Medical Advanced Care (C-MAC), bougie, Miller-Angle-Curved (MAC-COY), and standby tracheostomy tubes. Under aseptic conditions, a transtracheal block was administered using 2% lignocaine with a 22G Intravenous (IV) cannula after confirming air aspiration and negative blood aspiration. The patient was nasally intubated using a fibreoptic bronchoscope with a flexometallic (armoured) tube size of 7.0 mm, due to a decrease in mouth opening. The patient's vitals remained stable throughout the procedure. Post-surgery, inhalational agents were stopped, and after the patient exhibited spontaneous eye opening, adequate spontaneous breathing, and obedience to oral commands, he was extubated following administration of reversal agents Inj. Glycopyrrolate 0.008 mg/kg and Inj. Neostigmine 0.5 mg/kg.

Post-extubation, the patient experienced a significant drop in oxygen saturation to 60%. He was ventilated with a number four mask and 100% oxygen, which increased his saturation to 88%, while his blood pressure was 160/90 mmHg and end-tidal carbon dioxide (ETCO_2_) was 50 mm Hg. Despite these measures, secretions and pus emerged from his nose and oral cavity, and his saturation dropped again to 40%, leading to laryngospasm. Immediate interventions included administering 100 mg IV hydrocortisone, 2-3 puffs of salbutamol, and thorough oral suctioning. Despite these efforts, his oxygen saturation (SPO_2_) continued to fall, prompting attempts at reintubation with a C-MAC. However, airway edema and copious secretions obscured the vocal cords, necessitating an emergency tracheostomy.

**Figure 3:**
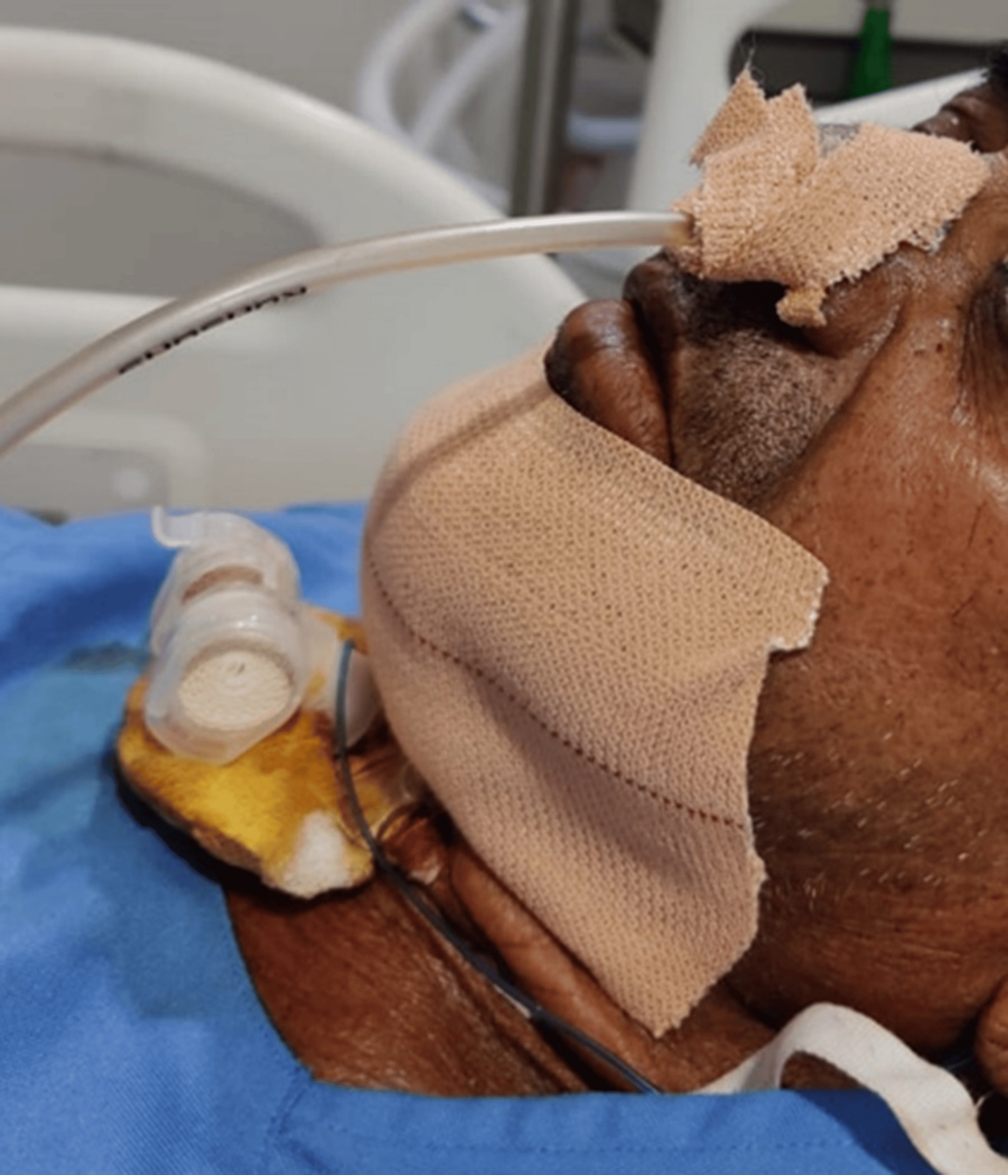
Patient in ICU with tracheostomy

The surgeons performed the tracheostomy using a 5.5-cuffed tube, which was successfully inserted and ventilated, raising the patient's SPO_2_ to 92%. Subsequently, the 5.5 cuffed ET tube was replaced with a 7.0 cuffed tracheostomy tube using a bougie. Post-procedure, bilateral airway entry was confirmed, the ETCO_2_ graph was checked, and SPO_2_ stabilized at 96%. Blood pressure rose to 168/98 mm Hg, ETCO_2_ normalized to 44, and heart rate increased to 120/min. The patient was administered 100 mg IV fentanyl and 4 mg IV vecuronium and maintained on 100% oxygen. Tracheostomy tube suctioning revealed no further secretions. The patient was then shifted to the ICU for ongoing observation and started on broad-spectrum antibiotics (Figure 3).

## Discussion

A difficult airway presents significant challenges for anesthesiologists, especially when performing mask ventilation or tracheal intubation. This difficulty is characterized by complications during tracheal tube insertion using a conventional laryngoscope, with difficult intubation defined as requiring three or more attempts or exceeding 10 minutes [1]. Factors contributing to such difficulty include anatomical variations such as obesity, high body mass index, advanced age, and higher Mallampati scores (grades three or four). A thyromental distance of less than 6 cm can further complicate the intubation process [2, 3].

Ludwig's angina is a particularly severe condition that complicates airway management due to its potential for rapid progression and severe airway obstruction [4]. This condition is characterized by gangrenous cellulitis of the neck that spreads through fascial planes, leading to significant edema and potential airway distortion [5]. Effective management of Ludwig's angina involves aggressive antibiotic therapy and, often, surgical drainage. Protecting the airway is paramount, given the risk of bilateral cervical lymph node swelling, drooling, dysphagia, neck tenderness, posterior tongue displacement, and restricted neck movement [6].

Preoperative assessment is crucial to identify potential difficulties in mask ventilation, laryngoscopy, and intubation [7,8]. An experienced team member should be involved in managing difficult airways, utilizing techniques such as mask ventilation, laryngoscopy, tracheal intubation, and placement of supraglottic devices [9]. For patients with head and neck infections or similar conditions, careful planning for reintubation and alternative airway management methods is essential [10,11]. Ensuring that alternate ventilation methods are immediately accessible and establishing a clear plan for scenarios where conventional methods are ineffective is key to safe airway management [7].

When an unanticipated difficult airway scenario unfolds, it is key to act in a structured and coordinated manner, with no unnecessary delays. Essential equipment for the management of the difficult airway must be rapidly accessed, and these tools should be logically organized. Once a difficult airway situation evolves, the risk of cognitive overload and stress-induced deterioration of decision-making and situational awareness increases. Hence, the design and setup of a dedicated difficult airway trolley (DAT) should, in addition to containing the adequate equipment, also ideally facilitate adherence to difficult airway algorithms to decrease the risk of human factor mistakes.

Difficult airway trolley (DAT) consists of Drawers 1-4:

Drawer 1 (plan A) intubation: Laryngoscopes, Macintosh sizes 3,4 and Millers sizes 2,3, videolaryngoscope blades of different types, Endotracheal tubes size 5.0, 6.0, 7.0, 8.0, Nasal Endotracheal tubes size 6.0, 7.0, stylet, Magill forceps.

Drawer 2 (plan B) oxygenation via a supraglottic airway device (SAD): Two different types of 2nd generation SADs. Sizes 3,4 and 5. Lubrication gel, adjuvants for flexible videobronchoscopic-guided intubation, e.g., endoscopy mask, breakaway oropharyngeal airway.

Drawer 3 (plan C) mask ventilation: Facemask sizes 3 and 4, oropharyngeal airway different sizes 7, 9, 10, 11 cm, Nasopharyngeal airway sizes 6.0, 7.0 and 8.0.

Drawer 4 (plan D) emergency invasive airway access: Emergency cricothyrotomy catheter set, Endotracheal tube size 6.0, scalpel blade 10.

## Conclusions

The current case report describes a severe and atypical presentation and management of Ludwig’s angina. Anesthesia management was particularly challenging due to several factors, including a difficult airway, reduced mouth opening, restricted neck movements, heavy jaw, double chin, airway edema, excessive secretions, and diminished visibility of the vocal cords. Extubation is crucial and of utmost importance in these cases. It should not occur until it has been determined that the patient can protect their airway and that the airway is patent. In cases with head and neck infections, extubation is equally important and critical. All preparation for reintubation and invasive techniques to secure the airway should be ready. The patient was successfully operated on for Ludwig’s angina, followed by an emergency tracheostomy in response to decreasing oxygen saturation. This case highlights the importance of prompt and effective multidisciplinary management in handling complex cases of Ludwig’s angina.
